# Boosting Therapeutic Effect of Turmeric, Coffee, and Chili Extracts Through Experimental Design and Encapsulation as Nanostructured Lipid Carriers for Novel Heath Supplements

**DOI:** 10.3390/plants14020236

**Published:** 2025-01-16

**Authors:** Pratchaya Tipduangta, Phennapha Saokham, Jutamas Jiaranaikulwanitch, Siriporn Okonogi, Chadarat Ampasavate, Kanokwan Kiattisin

**Affiliations:** 1 Department of Pharmaceutical Sciences, Faculty of Pharmacy, Chiang Mai University, Chiang Mai 50200, Thailand; pratchaya.t@cmu.ac.th (P.T.); phennapha.s@cmu.ac.th (P.S.); jutamas.jia@cmu.ac.th (J.J.); siriporn.o@cmu.ac.th (S.O.); chadarat.a@cmu.ac.th (C.A.); 2 Center of Excellence in Pharmaceutical Nanotechnology, Faculty of Pharmacy, Chiang Mai University, Chiang Mai 50200, Thailand

**Keywords:** curcumin, capsaicin, coffee, design of experiment (DoE), nanostructured lipid carriers (NLCs), antioxidant, anti-inflammation

## Abstract

This study investigates the potential synergistic effects of extracts from *Curcuma longa* (turmeric), *Coffea arabica* (Arabica coffee beans), and *Capsicum annuum* (chili peppers) in reducing oxidative stress and inflammation, which are associated with metabolic disorders such as obesity, diabetes, and cardiovascular diseases. Using a systematic design of experiment (DoE) optimization approach, an optimal extract ratio of 1:3:4 (turmeric: coffee: chili) was identified. The efficacy of the extract combination was assessed through various antioxidant assays, inhibition of inflammation-related gene expression, and safety testing via the 3-(4,5-dimethylthazolk-2-yl)-2,5-diphenyl tetrazolium bromide (MTT) assay. The extract combination showed higher antioxidant activity and comparable anti-inflammatory effects relative to each single extract. Additionally, the extract combination demonstrated effective activity compared with turmeric extract while using a lower concentration, resulting in reduced cytotoxicity. The optimized extract combination was successfully incorporated into nanostructured lipid carriers (NLCs) with a hydrodynamic diameter of 258.0 ± 10.2 nm, which effectively redisperses after the spray-drying process with increased diameter to 349.8 ± 49.6 nm. Under stress conditions, the stability of curcumin and capsaicin in dried-NLCs was maintained. In summary, the optimized extract-loaded NLCs formulation, achieved through a multistage approach, shows promise in mitigating oxidative stress and inflammation, suggesting its potential as a valuable daily dietary supplement.

## 1. Introduction

To address the serious threat caused by non-communicable diseases (NCDs) resulting from unhealthy lifestyles, pollution exposure, and stress, numerous spices have been identified for their pharmacological activities for NCDs prevention and treatment. The World Health Organization (WHO) recognizes NCDs as a significant public health concern. Conditions within the NCDs spectrum encompass obesity, diabetes, and high blood pressure, as well as hyperlipidemia, a precursor to stroke [1], and various cancers [2]. Obesity disrupts blood sugar regulation, leading to abnormal glucose levels in the bloodstream [3]. Additionally, it is associated with a decrease in high-density lipoprotein cholesterol (HDL-C) [4]. The consumption of fatty foods contributes to adipose tissue accumulation. Importantly, several studies have reported alterations in several inflammatory markers such as tumor necrosis factor-alpha (TNF-*α*), C-reactive protein (CRP), interleukins (IL-6, IL-18), resistin, and visfatin, leading to chronic low-grade inflammation in obese humans [5,6]. However, the association between abdominal and total obesity and various inflammatory markers is not fully understood [5]. The role of mild chronic inflammation in the pathophysiology of major causes of death, such as cardiovascular disease, is well established. Oxidative stress-induced cellular inflammation can down-regulate biomolecules, leading to organ dysfunction and various related diseases [4,7]. The consumption of fatty foods contributes to adipose tissue accumulation. Importantly, several studies have reported alterations in several inflammatory markers such as tumor necrosis factor-alpha (TNF-*α*), C-reactive protein (CRP), interleukins (IL-6 and IL-18), resistin, and visfatin, leading to chronic low-grade inflammation in obese humans. However, the association between abdominal and total obesity and various inflammatory markers is not fully understood [5]. The role of mild chronic inflammation in the pathophysiology of major causes of death, such as cardiovascular disease, is well established. Oxidative stress-induced cellular inflammation can down-regulate biomolecules, leading to organ dysfunction and various related diseases [7]. Consequently, the application of effective antioxidants from medicines or plants, assessed through in vitro and in vivo antioxidant tests, as well as clinical studies, is a promising avenue to mitigate inflammation and its associated diseases. Therefore, the antioxidant and anti-inflammatory activities of the optimized products are focused on in this study. The utilization of pharmacologically validated, edible herbal supplements should be warranted for their safety, efficacy, and intricate formulations, particularly in the prevention of metabolic syndromes. Understanding cellular signaling networks and their applications in combination therapies has been important in optimizing therapeutic interactions, commonly referred to as ’synergism’. In the treatment of complex diseases like cancer and metabolic syndromes, targeting a single pathway often fails to produce the desired therapeutic outcomes [8,9,10]. Extensive research and numerous theories have documented the quantification of synergy, as well as the benefits and drawbacks of administering compounds in combination [11,12].

The literature suggests that various plant extracts and herbs, such as turmeric (*Curcuma longa* L.), chili pepper (*Capsicum annuum*), and Arabica coffee (*Coffea arabica* L), have the potential to lower lipid levels in the blood [13,14,15,16]. These plants are commonly cultivated in Thailand, particularly in the northern and central regions. Turmeric and Arabica coffee bean extracts have garnered attention as health supplements readily available on the market, with turmeric extract being recognized for its weight loss properties [17,18]. Di Pierro et al. reported that administering 800 mg of curcumin for one month to healthy volunteers resulted in weight loss, improved body proportion, and reduced body mass index (BMI) [19]. Arabica coffee bean extract has been shown to lower lipid levels by stimulating beta-adrenergic receptor activity, reducing phosphodiesterase secretion, enhancing cyclic adenosine monophosphate secretion, and enhancing fat degradation in adipose tissue into triglycerides, free fatty acids, and adenosine triphosphate (ATP) [20]. Chili extract, known for its pain-relieving and anti-inflammatory properties when used topically, contains capsaicin which influences lipid breakdown in adipocytes [21]. This study demonstrated that applying chili extract containing 0.0075% capsaicin could reduce the liver-fat content of mice after 7 weeks [21]. Consequently, this research focuses on a combination of turmeric, chili, and coffee extracts due to their bioactive compounds influencing multiple pathways involved in inflammation, cancer, and lipid and carbohydrate metabolism. Therefore, the optimal combination of these three extracts could hold significant modulations in different major metabolic pathways and offer a convenient therapeutic approach such as medicine or supplements in the future.

Curcumin and capsaicin face stability challenges due to their rapid degradation through photolysis and oxidation [22]. Therefore, methods to enhance their stability are necessary. Nanostructured lipid carriers (NLCs) are a colloidal system comprising lipids (both solid and liquid lipids), emulsifying agent, and water. The incorporation of solid and liquid lipids in NLCs results in a less organized structure, thereby improving loading efficiency and stability [23,24]. Curcumin and capsaicin face stability challenges due to their rapid degradation through photolysis and oxidation [22]. Therefore, methods to enhance their stability are necessary. NLCs are a colloidal system comprising lipids (both solid and liquid lipids), an emulsifying agent, and water. The incorporation of solid and liquid lipids in NLCs results in a less organized structure, thereby improving loading efficiency and stability [23,24]. NLCs are widely employed for entrapping drugs and natural extracts, offering UV light protection, enhancing intestinal permeability, and improving the bioavailability of hydrophobic compounds [25,26,27]. Hence, we hypothesize that formulating turmeric, Arabica coffee bean, and chili extracts into NLCs will mitigate degradation, increase solubility, enhance absorption, and alleviate stomach irritation.

Therefore, this research aimed to assess the feasibility of employing a blend of turmeric, Arabica coffee bean, and chili extracts to maximize their pharmacological efficacy by identifying optimal ratios and concentrations for the development of health supplement via NLCs. In addition, the release behavior of the formulation was studied. The stability of the prototype products under varied conditions and the absorption behavior of chemical components in turmeric, coffee, and chili was investigated as a potential prototype for inclusion in daily supplement regimens.

## 2. Results and Discussion

### 2.1. Optimized Mixed Extract Concentrations and Ratio by Design of Experiment Approach

Obesity-related diseases, such as cardiovascular diseases, diabetes, cognitive dysfunction, and cancer, are related to oxidative stress in the body. Previous research reported that natural antioxidants can reduce oxidative stress in obesity [28,29,30]. Polyphenol antioxidant compounds, including curcumin, quercetin, and catechins, exhibited positive effects against metabolic disorders [31]. Therefore, an increase in antioxidants in the body may prevent and reduce the risk of various diseases. The antioxidant activity of mixed extracts was assessed using two different methods: 2,2-diphenyl-1-picrylhydrazyl radical scavenging activity (DPPH-RSA) and lipid peroxidation inhibition assays. The DPPH-RSA assay is employed to assess stable free-radical scavenging activity by electron transfer [32]. The lipid peroxidation inhibition by ferric thiocyanate method is used to evaluate the ability of the sample to inhibit peroxyl radicals in the early stage of lipid peroxidation [33]. The appropriate concentration ratio of turmeric, chili, and coffee extracts that achieved the highest antioxidant activity was determined using the Design of Experiment approach [28,29,30]. Polyphenol antioxidant compounds, including curcumin, quercetin, and catechins, have exhibited positive effects against metabolic disorders [31]. Therefore, an increase in antioxidants in the body may prevent and reduce the risk of various diseases. The antioxidant activity of mixed extracts was assessed using two different methods: DPPH-RSA and lipid peroxidation inhibition assays. The DPPH-RSA assay is employed to assess stable free radical scavenging activity by electron transfer [32]. The lipid peroxidation inhibition by ferric thiocyanate method is used to evaluate the ability of the sample to inhibit peroxyl radicals in the early stage of lipid peroxidation [33]. The appropriate concentration ratio of turmeric, chili, and coffee extracts that achieved the highest antioxidant activity was determined using the Design of Experiment approach.

The lower limit and upper limit of the extract concentrations in the full factorial design runs experiments were selected through our preliminary DPPH-RSA assay (Table S1). The concentration ranges of turmeric extract, Arabica coffee bean extract, and chili extract were calculated by multiplying 10 to 100 times each extract’s half maximal inhibitory concentration (IC_50_) obtained from the DPPH-RSA assay. The lower and upper concentration limits of each extract used in the DoE experiments are shown in Table S2. The turmeric extract concentration exhibited a range from 140 to 1400 µg/mL. In the case of coffee extract, the concentration was between 6000 and 60,000 µg/mL. As for chili extract, its effective concentration spans between 800 and 8000 µg/mL. Within these concentration ranges, all extracts showed antioxidant properties. The results of the DPPH-RSA assay and lipid peroxidation inhibition assay are reported in Table 1. The relationships between turmeric extract, chili extract, and coffee extract concentrations and antioxidant properties were calculated via linear regression.

The DPPH-RSA assay revealed that, among all the extracts, only the concentration of turmeric extract significantly impacted the percentage inhibition (*p*-value < 0.05). The percentage inhibition in the DPPH-RSA assay was found to be dependent on the concentration of turmeric extract, as illustrated in Figure S1A. By excluding terms that were not statistically significant in the relationship (*p*-value > 0.05), as indicated in Table S3, it becomes possible to evaluate the appropriateness of the lack-of-fit equation and the tendency of the relationship to be polynomial. The relationship between the concentration of turmeric extract in the mixed extract and percentage inhibition, as tested by the DPPH-RSA method, was identified as a linear relationship (*p*-value of curvature and lack of fit > 0.05), as shown in Equation (1):

% inhibition = 80.38 + 0.01 × [Turmeric extract concentration](1)

**Figure 1:**
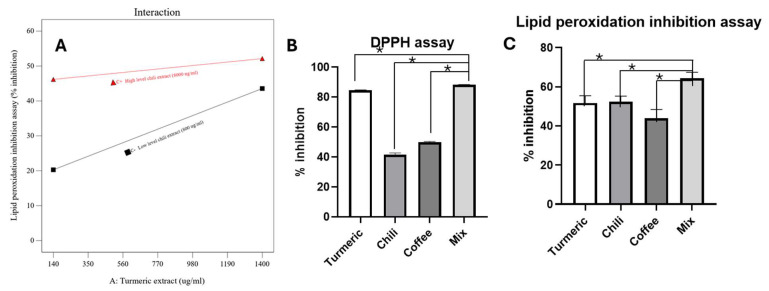
(**A**) Relationship between the combination effect of turmeric and chili extract concentrations and % lipid peroxidation inhibition. (**B**) Percentage inhibition in DPPH-RSA assay for turmeric (1400 µg/mL), coffee (6000 µg/mL), chili (8000 µg/mL), and their mixture (1400:6000:8000 µg/mL). (**C**) Percentage inhibition in lipid peroxidation assay for turmeric (1400 µg/mL), coffee (6000 µg/mL), chili (8000 µg/mL), and their mixture (1400:6000:8000 µg/mL). Asterisk (*) indicates statistical significance (*p*-value < 0.05, ANOVA).

The concentrations of turmeric and chili extracts significantly demonstrated positive effects on percentage inhibition in the lipid peroxidation inhibition assay, as depicted in Figure S1B,C. The synergistic effect of the turmeric and chili extract concentrations on percentage inhibition in the lipid peroxidation inhibition assay is evident in Figure 1A. When interactions between two extracts were observed, black and red lines representing low and high levels, respectively, were added to the graph to highlight the trends in their interaction. Notably, increasing the concentration of turmeric extract in the mixed extracts, particularly when the concentration of chili pepper extract was high (8000 μg/mL), increased the ability to inhibit lipid oxidation compared with instances where the chili pepper extract concentration was low (800 μg/mL). Upon excluding non-significant terms, the relationship between the percentage inhibition in lipid peroxidation and the concentrations of turmeric and chili extracts can be described linearly in Equation (2). However, the lack-of-fit *p*-value in this model was less than 0.05 due to the interference of the three-factor co-influence involving the concentrations of turmeric, coffee, and chili extracts.

% inhibition = 12.87 × + 0.01 × [Turmeric extract concentration] + 0.003 × [Chili extract concentration](2)

Based on the correlation, it could be interpreted that an increase in turmeric concentration led to higher antioxidant activity across all assays. The antioxidant mechanisms of turmeric are attributed to the presence of curcuminoids, which include free-radical scavenging and inhibition of lipid peroxidation [34]. The inhibition of lipid peroxidation, which represents the antioxidant mechanism in non-aqueous conditions, corresponded to higher concentrations of chili extract. The integration of an appropriate ratio and concentration of these three components influenced the antioxidant activity in aqueous, semi-polar, and nonpolar biosystems. The optimization process aimed to achieve the highest percentage inhibition in both the DPPH-RSA and lipid peroxidation inhibition assays. With a desirability value of 1.000, the prediction concentration ratio of the three extracts was 1400:6000:8000 µg/mL.

This ratio was subsequently validated by assessing the percentage inhibition, with the results detailed in Table 2. The percentage inhibition in the DPPH-RSA assay for both predicted and experimental values fell within an acceptable range. However, a deviation between the experimental and predicted values was observed in the percentage inhibition of lipid peroxidation, likely attributable to the co-influence of other extract concentrations. The results of DPPH-RSA assay and lipid peroxidation assay of turmeric (1400 µg/mL), coffee (6000 µg/mL), chili (8000 µg/mL), and their mixture (1400:6000:8000 µg/mL) are illustrated in Figure 1B,C. The DPPH-RSA assay indicated a marginal increase in inhibition percentage for the mixed extract relative to turmeric extracts. The percentage inhibition of the mixed extract was significantly higher than that of both the coffee and chili extracts individually (Figure 1B). Conversely, the lipid peroxidation assay revealed a marked synergistic effect in the mixed extract compared with the individual components (Figure 1C). The mixed extract showed a statistically significant difference (*p* < 0.05) from each individual extract in both the DPPH-RSA and lipid peroxidation assays. The mixed extract can potentially inhibit electron transfer from DPPH radicals by completing the electron pair, thereby neutralizing the free radical [35]. It may also prevent lipid decomposition by inhibiting the oxidation of linoleic acid, effectively stopping the chain reaction [36]. Synergistic effects of mixed extracts or plant extract with synthetic antioxidant have been widely reported in the literature [37]. The oxidation process involves a complex set of mechanisms, and mixtures of compounds, whether phytochemicals or synthetic, often exhibit greater bioactivity than individual compounds. This is because a combination of bioactive compounds can simultaneously influence multiple targets and/or antioxidant mechanisms. Regeneration mechanism had been hypothesized to explain the mechanisms of synergism and antagonism. This depends on the chemical structure of molecules and the possible formation of stable intermolecular complexes [37,38].

### 2.2. MTT Assay of RAW264.7 Cells

The MTT assay was used to identify the non-cytotoxic concentrations of coffee extract, turmeric extract, chili extract, and their combination. Table 3 presents the IC_50_ values of cytotoxicity for each individual extract and the mixed extract. Among the extracts, turmeric exhibited the lowest IC_50_ value, indicating that it was the least safe for use at high concentrations. This was followed by the mixed extract, chili extract, and coffee extract, respectively. The mixed extract at concentrations of 1400:6000:8000 µg/mL appeared to reduce the cytotoxicity of the turmeric extract.

### 2.3. Effect of Coffee Extract, Turmeric Extract, Chili Extract, and Mixed Extract on the Expression of Genes Involved in Inflammation

**Figure 2:**
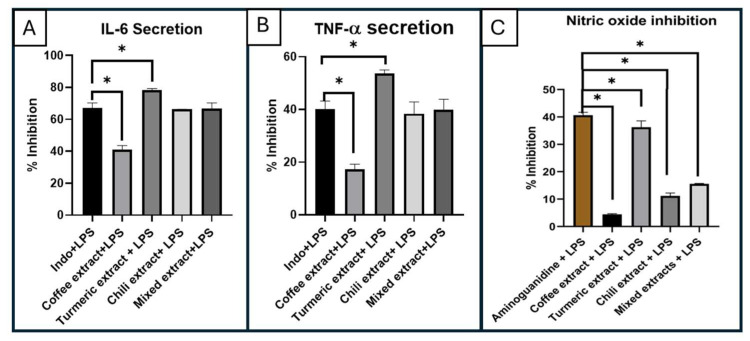
Percentage inhibition of (**A**) IL-6 secretion and (**B**) TNF-α secretion of the cells with coffee extract, turmeric extract, or chili extract, or mixed extract with LPS, and Indomethacin with LPS as the standard. (**C**) Percentage inhibition of nitric oxide secretion of coffee extract, turmeric extract, or chili extract, or mixed extract with LPS, and Aminoguanidine with LPS as the standard. Asterisk (*) indicates statistical significance (*p*-value < 0.05, ANOVA).

The percentage inhibition of inflammatory genes, including IL-6 and TNF-α, were evaluated across various conditions: cells without lipopolysaccharide (LPS), cells added with LPS (50 ng/mL), cells with single extract (at a concentration of 10 μg/mL), cells with mixed extract (also at a concentration of 10 μg/mL), cells with single extract and LPS, cells with mixed extract and LPS, and cells with Indomethacin and LPS (at a concentration of 10 μg/mL) serving as the standard (Figure 2A,B). The gene expression results of IL-6 and TNF-α for coffee bean extract, turmeric extract, chili extract, and mixed extract resembled those of untreated cells.

In Figure 2A, the coffee extract exhibited the lowest percentage inhibition of IL-6 secretion, at 41.12 ± 2.42%. The chili extract, mixed extract, and indomethacin showed similar percentage inhibition values of IL-6 secretion, with values of 66.23 ± 0.17%, 66.68 ± 3.54%, and 66.96 ± 3.24%, respectively. The turmeric extract demonstrated the highest percentage inhibition of IL-6 secretion at 78.26 ± 1.04%. The percentage inhibition of TNF-α secretion in Figure 2B followed a consistent trend with IL-6 secretion. Coffee extract exhibited the lowest percentage inhibition of TNF-α secretion, at 17.30 ± 1.93%, followed by chili extract, mixed extract, and indomethacin, with values of 38.41 ± 4.44%, 39.85 ± 4.00%, and 40.18 ± 2.97%, respectively. Turmeric extract showed the greatest percentage inhibition of TNF-α secretion, at 53.61 ± 1.43%. The mixed extract exhibited comparable percentage inhibition values of both IL-6 and TNF-α secretion to the indomethacin positive control. However, turmeric extract was the strongest inhibitor of both IL-6 and TNF-α secretion compared with the other extracts and indomethacin.

### 2.4. Anti-Nitric Oxide Secretion Study

The result regarding the anti-nitric oxide secretion effect revealed significant findings when coffee bean extract, turmeric extract, chili extract, or mixed extracts were added at a concentration of 10 μg/mL to cells with an LPS concentration of 50 ng/mL (Figure 2C). A comparison of the percentage inhibition of nitric oxide secretion indicated that turmeric extract + LPS exhibited the highest inhibitory effect at 36.25% ± 2.31, followed by mixed extract + LPS, chili extract + LPS, and arabica coffee bean extract + LPS, with values of 15.56% ± 0.16, 11.18% ± 1.04, and 4.44% ± 0.26, respectively. However, all extracts and mixed extracts showed significantly lower percentage inhibition than the Aminoguanidines + LPS control group. Consequently, turmeric extract was the most promising candidate for anti-inflammatory action via nitric oxide suppression. Adipose tissue in obesity upregulates the expression of inflammatory cytokines, including IL-1β, IL-6, IL-8, IL-10, IL-12, and TNF-α [6,39,40]. This research focuses on IL-6 and TNF-α. The secretion of IL-6 and TNF-α affect body weight, lipid metabolism, and insulin resistance [41,42,43]. These inflammatory cytokine levels can serve as a tool for diagnosing obesity. Therefore, the extracts that can inhibit the expression of these cytokines have the potential to reduce inflammation in the body. Previous research has reported the anti-inflammatory activity of curcumin, capsaicin, and caffeine. However, research related to anti-inflammatory activity of mixed extracts is limited. Curcumin has anti-inflammatory effects by regulating inflammatory signaling pathways and inhibiting the production of inflammatory cytokines. It decreases the levels of IL-1, IL-1β, IL-6, IL-8, IL-17, IL-27, TNF-α, inducible nitric oxide synthase (iNOS), and nitric oxide [44,45,46]. Capsaicin decreases the levels of IL-8, IL-6, TNF-α, and NO [47,48]. Another study reported anti-inflammatory activities of a combination of silibinin and capsaicin. The results showed that the combination strongly inhibited NO, TNF-α, IL-6, and COX-2 [49]. Caffeine can decrease IL-1β, IL-6, TNF-α, and iNOS [50,51]. The results showed that the combination strongly inhibited NO, TNF-α, IL-6, and COX-2 [49]. Caffeine can decrease IL-1β, IL-6, TNF-α, and iNOS [50,51].

Based on the results of the in vitro antioxidant test, the optimal ratio for the synergistic effect of turmeric extract, coffee extract, and chili extract was determined to be 1:4.28:5.71. However, considering formulation feasibility, the need to minimize chili extract irritation during preparation, and the goal of maximizing health benefits, adjustments were made. Turmeric extract, which demonstrated strong bioactivity—including a high percentage inhibition in the DPPH-RSA assay and potent inhibition of IL-6 secretion, TNF-α secretion, and nitric oxide production—was slightly increased while maintaining fixed proportions of coffee and chili extracts. Consequently, the 1:3:4 ratio of curcumin extract, coffee extract, and chili extract was adopted as an optimal balance between practical formulation requirements and enhanced bioactivity. In addition, this ratio has a positive effect on in vitro anti-inflammatory activity. However, considering the potential for severe mucosal irritation and stomach upset due to the inclusion of a high level of capsaicin in the formulation, the content of turmeric extract, coffee extract, and capsaicin extract was also regulated based on the maximum safe human daily dose of capsaicin. The EU recommends a safe dietary capsaicin content ranging from 0.77 to 2.64 mg/day [52,53]. Consequently, we selected a nominal capsaicin dose of 0.9 mg per day to minimize irritation to capsaicin intolerance. The analysis of capsaicin content in the chili extract used in this study indicated that it contained 13% of the extract. To achieve a capsaicin content of 0.9 mg, the chili extract must be used at a quantity of 7.08 mg. Calculating the three mixtures in accordance with the 1:3:4 ratio for a daily dose revealed a composition of various substances, as detailed in Table 4. Consequently, the dosage of the mixed extract formulated into NLCs was determined to be 14.16 mg per day.

### 2.5. Development and Characterization of NLCs

Preliminary experiments indicated that the loading efficiency of Arabica coffee bean extract in NLCs was limited, possibly due to the hydrophilic nature of caffeine. To enhance this efficiency, NLCs were separately prepared with concentrations of 0.1% *w*/*w*, along with turmeric extract and chili extract, at concentrations of 0.02% and 0.08% *w*/*w*, respectively. These NLCs were then mixed with Arabica coffee bean extract in an optimal ratio determined through Design of Experiments. Before mixing, the hydrodynamic diameter and zeta potential of the turmeric and chili extract NLCs were individually measured and compared with those of the final mixture. The results showed significant increases in hydrodynamic diameter and zeta potential between the blank and mixed NLCs at a 95% confidence interval (*p*-values of 0.0002 and 0.0243, respectively), while the polydispersity index (PDI) values did not change significantly (Figure S2A–C).

**Figure 3:**
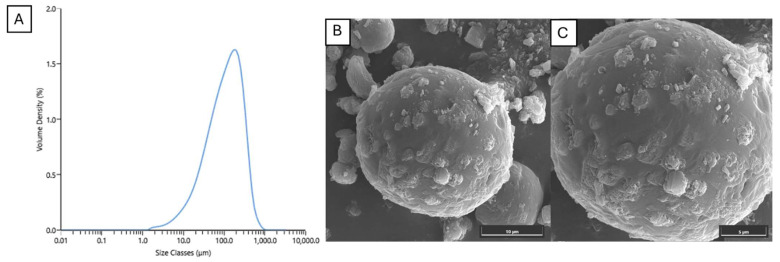
(**A**) Particle size distribution of spray-dried NLC particles (**B**,**C**) SEM morphology of NLC particles after spray drying process.

The mixture of loaded NLCs exhibited a hydrodynamic diameter of 258.0 ± 10.2 nm, a PDI of 0.576 ± 0.031, and a zeta potential of −29.9 ± 1.0 mV. After spray-drying, the dried NLCs had a monodispersed size of 128.67 ± 4.93 μm with a span of 2.553 ± 0.06 (Figure 3A). Upon redispersion in deionized (DI) water, the hydrodynamic diameter and zeta potential of the loaded NLCs increased to 349.8 ± 49.6 nm, with a PDI of 0.605 ± 0.111 and a zeta potential of −29.7 ± 1.5 mV. Although there was a statistically significant increase in hydrodynamic diameter after redispersion compared with the mixed NLCs (*p*-value less than 0.05), both still fell within the nano size range.

The mannitol in the dried NLCs, which dissolved during redispersion, may have affected the DLS measurement results by altering the refractive index and viscosity of the dispersant. The presence of mannitol can influence the scattering intensity and the Brownian motion of particles, thereby impacting the measured particle size and zeta potential [54]. Considering the SEM images, it was revealed that the spherical spray-dried NLCs consisted of multiple loaded NLCs bound together with mannitol (Figure 3B,C). Therefore, it is suggested that the spray-drying process with mannitol effectively preserved the NLC structure and prevented agglomeration.

### 2.6. Stability Study of NLC Formulation Under Stress Conditions

**Figure 4:**
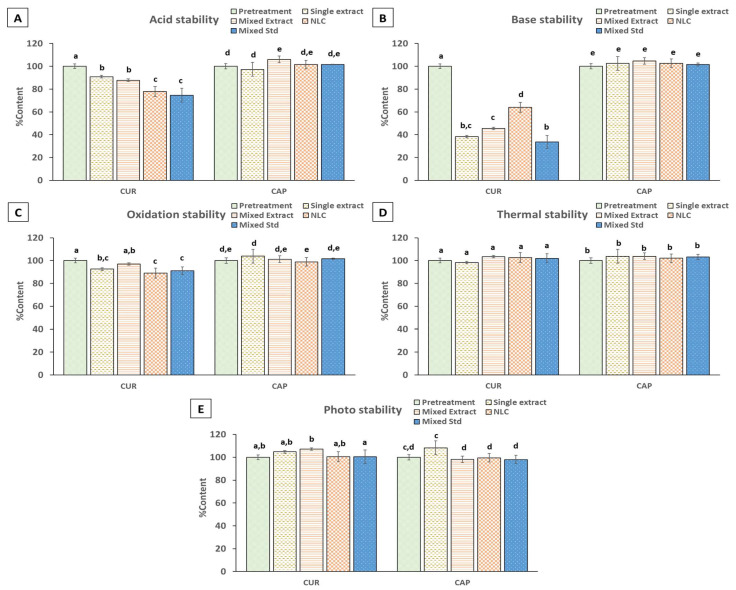
Stability of single extract, mixed extract, NLCs, and mixed standard under different stress conditions: (**A**) acid stability, (**B**) base stability, (**C**) oxidation stability, (**D**) thermal stability, and (**E**) photo stability. (ANOVA, the same letter indicates a *p*-value > 0.05 between group).

In this study, curcumin and capsaicin were chosen as markers to assess the stability properties of the formulation because their quantity in the formulation was sufficient to be analyzed and the substance had unstable properties in various conditions, such as acid, basic, and oxidation conditions [22,55,56]. Meanwhile, chlorogenic acid and caffeine were not used in the stability studies because they were present in small amounts in the formulation. The mixed extracts were formulated in NLCs and their stability was compared with individual extracts and the mixed standard, which consisted of curcumin, capsaicin, and chlorogenic acid at the ratio of 1:1:1. Even under strongly acidic conditions, curcumin in samples was slightly degraded, and curcumin in both single and mixed extract were more stable than those standard mixtures. The degradation of curcumin in the NLC formulation was insignificantly different compared with that of the mixed extract (Figure 4A). Conversely, capsaicin in the chili extract, mixed extracts, mixed standard, and NLCs remained stable under acidic conditions (Figure 4A), with no significant decrease from the pretreatment at 100% content. However, curcumin exhibited instability under basic pH values across all samples, with a noticeable decrease observed from pretreatment at 100% content (Figure 4B). Interestingly, the curcumin content in NLCs was significantly higher than that in other samples. This result shows that NLCs can prevent the degradation of curcumin in basic conditions [56]. Similar to acidic conditions, capsaicin did not exhibit a significant decrease in content between chili extract, mixed extracts, mixed standard, and NLCs as compared with pretreatment (Figure 4B). Under oxidative stress conditions, curcumin exhibited slight instability, leading to a decrease in turmeric extract. However, curcumin in the mixed extract exhibited no significant difference from pretreatment (Figure 4C). Capsaicin remained stable under oxidative stress conditions in chili extract, mixed extracts, mixed standard, and NLCs (Figure 4C). Under thermal stress conditions, curcumin levels showed no significant difference among turmeric extract, mixed extract, mixed standard, and NLCs, with the single extract exhibiting the lowest amount of curcumin (Figure 4D). Similarly, capsaicin remained stable under thermal stress conditions in all tested samples. Figure 4E illustrated the stability of both curcumin and capsaicin in all tested samples under photo stress conditions. There were no significant reductions from the pretreatment. In summary, curcumin in NLCs formulation enhanced stability under basic pH values. Conversely, capsaicin remained stable under all stress conditions, with no significant impact observed from NLCs preparation.

### 2.7. Release Study and Kinetic Data Model Fitting

**Figure 5:**
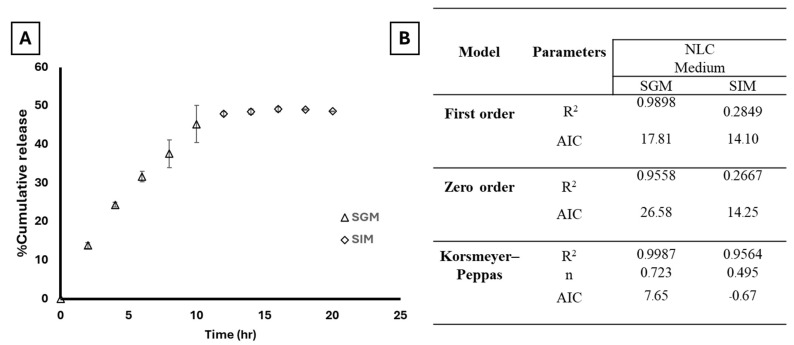
Curcumin release profile of NLCs formulation in (**A**) simulated gastric medium (SGM) and simulated intestinal medium (SIM) (**B**) Correlation coefficient (R^2^), n value of curcumin release, and AIC obtained from fitting kinetic model.

Curcumin was used as a marker to monitor the release behavior of NLCs due to its potent antioxidant and anti-inflammatory properties and comparatively lower stability compared with capsaicin. Figure 5A illustrates the release profile of curcumin from NLCs, in simulated gastric medium (SGM) and simulated intestinal medium (SIM). In SGM, the NLC formulation released approximately half of its curcumin content within 10 h in SGM. Subsequently, in SIM, less than 5% of curcumin was released within 10 h. In our experiment, polymers with molecular weights greater than 15,000 Da were trapped in the dialysis sack. This indicates that the observed amount of curcumin was freely available from the formulations, ready to be absorbed through gastric or intestinal phospholipid membranes.

Various kinetic models were employed to describe the release behavior of curcumin from NLC, with the coefficients (R^2^), Akaike information criterion (AIC), and parameters reported in Figure 5B. Notably, the fit of NLC release in SIM to both zero-order and first-order models was poorer, though it slightly favored the first-order model. The Korsmeyer–Peppas model exhibited good fit with NLCs, attributed to it containing more variables for fitting compared with the zero-order and first-order models, thus resulting in improved data fitting and higher R^2^ values and low AIC [57]. The n value obtained from the Korsmeyer–Peppas model determines the mechanism of curcumin release from NLCs formulation. A value greater than 0.5 suggests anomalous transport, which is a combination of Fickian diffusion and Case-II transport. Values closer to 1 indicate predominance by Case-II transport, while those closer to 0.5 indicate dominance by Fickian diffusion. A value greater than 0.5 suggests anomalous transport, which is a combination of Fickian diffusion and Case-II transport. Values closer to 1 indicate a predominance of Case-II transport [58]. Several novel drug delivery systems have been developed to incorporate sustained-release characteristics, thereby enhancing the bioavailability of curcumin in humans. Among these strategies, nanoparticle-based delivery systems have demonstrated superior potential in improving curcumin bioavailability, leading to enhanced therapeutic efficacy as evidenced by clinical trials [59,60]. The concomitant administration of other active compounds has been also demonstrated to enhance curcumin bioavailability in both animal and human studies, with piperine serving as a notable example due to its potent inhibition of drug metabolism and glucuronidation [61]. Future investigations into the bioavailability of curcumin and capsaicin in vivo are warranted to elucidate the effects of the studied mixed extract formulated in the NLC system.

## 3. Materials and Methods

### 3.1. Materials

Turmeric powder extract containing 66.20% curcumin was purchased from Welltech Biotechnology Co., Ltd. (Bangkok, Thailand). Arabica green coffee bean liquid extract containing chlorogenic acid (0.51%) and caffeine (0.05%) was purchased from Specialty Natural Products Public Company Limited (Chon Buri, Thailand). Chili crude extract containing 13.82% capsaicin was purchased from Thai-China Flavours and Fragrances Industry Co., Ltd. (Nonthaburi, Thailand). D-mannitol (AR grade) was purchased from Kemaus, Australia.

Pepsin, lipase, bile extract, pancreatin, calcium chloride, and sodium acetate were purchased from Sigma-Aldrich (Darmstadt, Germany). Sodium chloride (NaCl) was purchased from Loba Chemie (Mumbai, India). Hydrochloric acid, methanol, acetic acid, dimethylsulfoxide (DMSO), and acetonitrile were purchased from RCI Labscan (Bangkok, Thailand). Formic acid was purchased from Fisher Scientific (Waltham, MA, USA). 2,2-Diphenyl-1-picrylhydrazyl (DPPH) was purchased from Fluka (Buchs, Switzerland). Linoleic acid and 2,4,6-Tris(2-pyridyl)-s-triazine (TPTZ) were purchased from Sigma (St. Louis, MO, USA). 2,2′-Azobis-(2-amidinopropane dihydrochloride) (AAPH) was purchased from Merck (Darmstadt, Germany). Sodium acetate (CH_3_COONa), ferrous chloride (FeCl_2_), and ferrous sulfate (FeSO_4_) were purchased from Sigma-Aldrich, Steinheim, Germany. β-actin, IL-6, and TNF-α were purchased from Humanizing Genomics Macrogen (Seoul, Korea).

The standard curcumin and capsaicin were purchased from the Department of Medical Sciences Reference Standards, Ministry of Public Health, Nonthaburi, Thailand. Glyceryl monostearate, acetyl alcohol, and isopropyl myristate were purchased from Chanjao Longevity Co., Ltd. (Bangkok, Thailand).

### 3.2. Optimization of Ratios and Concentrations of Turmeric, Arabica Coffee Bean, and Chili Extracts on Antioxidant Properties

Design-Expert software version 10 (Stat-Ease, Minneapolis, MN, USA) was utilized to optimize the ratios and concentrations of turmeric, Arabica coffee bean, and chili extracts for the extract mixture required in formulations. The concentration ranges of turmeric extract, Arabica coffee bean extract, and chili extract used for optimizing the appropriate concentrations by DOE were calculated by multiplying 10 to 100 times each extract’s IC_50_ obtained from the DPPH-RSA assay. The concentration ranges for the extracts were as follows: turmeric extract ranged from 140 to 1400 µg/mL, Arabica coffee bean extract ranged from 6000 to 60,000 µg/mL, and chili extract ranged from 800 to 8000 µg/mL. Various concentrations of each extract were prepared in ethanol and arranged into seventeen runs, as detailed in Table 1. Full factorial design was employed to identify the effect of each extract concentration in the mixtures. The DPPH-RSA assay and lipid peroxidation inhibition assay were employed to assess antioxidant properties. Subsequently, the Design-Expert program was employed for analysis of the optimal concentrations with the highest activity for further investigation.

#### 3.2.1. DPPH Free Radical Scavenging Activity Assay

This experiment followed the procedure outlined in the adapted methodology of Brem et al. [62]. An extract solution, with concentrations ranging from 0.0 to 1.0 mg/mL, was prepared, and 20 µL of this sample was mixed with 180 µL of DPPH reagent (0.033 mg/mL). The mixture was then incubated and protected from light at an ambient temperature for 30 min. Absorbance readings were taken at 520 nm using a microplate reader (SpectraMax M3, Molecular Devices, San Jose, CA, USA). Trolox served as the positive control in this experiment. The ability of sample to scavenge DPPH radicals was expressed as the percent inhibition (% inhibition) and was calculated using Equation (3).

Inhibition = [(Ac − An) − (As − Ab)/(Ac − An)] × 100 (3)

where Ac is the absorbance of the DPPH reagent, An is the absorbance of the solvent of DPPH, As is the absorbance of the sample mixed with DPPH, and Ab is the absorbance of the sample mixed with solvent.

#### 3.2.2. Lipid Peroxidation Inhibition Assay

This experiment followed the methodology outlined in [63]. Stock solutions of individual extracts and mixed extracts (concentration 50.0 mg/mL) were dissolved in 50% DMSO. Subsequently, the extract solution (150 µL) was combined with DI water (100 µL), 20 mM phosphate buffer solution with a pH of 7.0 (350 µL), 1.3% *v*/*v* linoleic acid (350 µL), and AAPH in 20 mM phosphate buffer saline (PBS) (pH 7.0) (50 µL). The sample was then incubated at 50 °C for 4 h. Subsequently, the sample (5 µL) was mixed with 10% ammonium thiocyanate (5 µL) and 20 mM ferrous chloride (5 µL). Afterward, 75% methanol (185 µL) was added to the sample. The absorbance of the sample was measured at 500 nm after 3 min of incubation using a Genios Pro microplate reader (Tecan, Crailsheim, Germany). Trolox served as a positive control. The percentage inhibition was subsequently determined in accordance with Equation (3).

### 3.3. Anti-Inflammatory Activity in Murine Macrophage

#### 3.3.1. Cell Culture

The RAW264.7 cells (Murine macrophage cells) were purchased from PromoCell (Heidelberg, Germany). Dulbecco’s modified eagle medium (Gibco, Waltham, MA, USA) supplemented with 10% heat-inactivated calf serum (HyClone, Logan, UT, USA) and 1% penicillin (100 U/mL)–streptomycin (100 μg/mL) (Gibco, New York, NY, USA) was used as culture media for the cells and incubated at 37 °C with 5% CO_2_ in a CO_2_ incubator (Thermo Fisher Scientific, Oxford, UK).

#### 3.3.2. Cytotoxicity Test

Different concentrations of extracts were applied to the cells and then incubated at 37 °C with 5% CO_2_ in a CO_2_ incubator for 24 h. Coffee extract and chili extract were used at concentrations ranging from 156 to 2500 μg/mL. Turmeric extract and mixed extracts were used at concentrations ranging from 10 to 156 μg/mL. The 3-(4,5-dimethylthazolk-2-yl)-2,5-diphenyl tetrazolium bromide or MTT (Sigma-Aldrich, St. Louis, MO, USA) assay were used to determine cytotoxicity of the sample. The sample was measured at 570 nm. The percent cell viability was calculated [(Absorbance sample-treated cells/Absorbance untreated cells) × 100] and reported as IC_50_. The experimental procedures were conducted in triplicate (n = 3).

#### 3.3.3. Nitric Oxide Inhibitory Test

After the pre-incubation of RAW264.7 cells (1.5 × 10^5^ cells/mL) with test samples at non-toxic concentrations under a 37 °C, 5% CO_2_ condition for 24 h, lipopolysaccharide (LPS) from *Escherichia coli* (10 µg/mL) was added to the sample and then incubated for 24 h. The amounts of nitrite, an indicator of nitric oxide (NO) production, were measured using Griess reagent (1% sulfanilamide) (Sigma-Aldrich, Hong Kong, China) and 0.1% naphthylethylenediamine dihydrochloride (Sigma-Aldrich, Bangalore, India) in 2.5% phosphoric acid. Briefly, cell culture medium (100 μL) and Griess reagent (100 μL) were mixed and incubated at room temperature for 10 min. The absorbance of the sample was measured at 540 nm using a microplate reader (EZ Read 2000 Microplate Reader, Biochrom, Cambridge, UK). Aminoguanidine served as a positive control. Then, the percent NO inhibition was calculated.

#### 3.3.4. Inflammatory-Related Gene Expression

The RAW264.7 cells were cultured and treated with various concentrations of extract in a 12-well plate at 37 °C with 5% CO_2_ for 24 h. After incubation overnight, the LPS (50 ng/mL) was added and then further incubated for 24 h. A ribonucleic acid (RNA) extraction kit (PureLink RNA Mini Kit, Invitrogen, Waltham, MA, USA) was used to extract the total RNA from the treated cells. The first-strand complementary DNA (cDNA) was synthesized using a reverse-transcription kit (iScript cDNA Synthesis kit, Bio-Rad, Hercules, CA, USA) and polymerase chain reaction (PCR) was conducted using a kit (Mytaq Red Mix, Bioline, UK) based on the previous research [64]. cDNA was synthesized with a reverse-transcription–polymerase chain reaction (RT-PCR) kit using a specific primer (Humanizing Genomics Macrogen, Seoul, Republic of Korea) and amplified using a thermal cycler (Heal Force Classic, K960-Thermo Cycler, Hefei, China) and the PCR profiles were 30 cycles. The PCR products were then analyzed on 1.5% agarose gel (Invitrogen, Waltham, MA, USA) via the electrophoresis process (Bio-Rad, Hercules, CA, USA). The sample was visualized by gel staining (Visafe Green, Gel Stain, Vivantis, Selangor Darul Ehsan, Malaysia) and the densities of RT-PCR products were measured using a Gel Documentation and System Analysis machine (Transilluminator Boi View, Bio Step, Jahnsdorf, Germany). The inflammatory-related gene expressions including IL-6 and TNF-α were calculated compared with β-actin. Then, the percent cytokine inhibition was calculated.

### 3.4. NLCs Preparation

Turmeric extract, Arabica coffee bean extract, and chili extract were individually encapsulated into nanostructured lipid carriers (NLCs), each containing 0.1% (*w/w*) of curcumin, chlorogenic acid (*w*/*w*), and capsaicin (*w*/*w*). The NLC formulation comprised a lipid mixture with surfactants, specifically 0.9% stearic acid, 0.1% oleic acid, 1.54% Tween 20, and 0.96% Span 20, which were selected to achieve an optimal hydrophilic–lipophilic balance (HLB). The choices of liquid and solid lipids, surfactant combinations, and NLC preparation methods were modified from Anantaworasakul et al. [65]. The lipid phase and extracts were heated to 70 °C under controlled stirring, while the aqueous phase containing non-ionic surfactant was heated to 75 °C. Subsequently, both phases were combined to form a pre-emulsion, which underwent particle size reduction using a high-shear homogenizer at 10,000 rpm for 10 min (Ultra-Turrax^®^ T25, IKA-Werke GmbH & CompanyKG, Staufen, Germany). The NLCs of each extract were mixed in the optimized ratio prior to spray-drying with 20% *w*/*w* mannitol. The NLCs were transformed into powder using a laboratory-scale Büchi B290 mini spray-dryer (Büchi Labortechnik AG, Flawil, Switzerland). The inlet temperature was fixed at 100 °C and 100% air flow aspiration. Feed solution atomization utilized a 0.7 mm nozzle with an airflow of 473 L/h and a feed rate setting of 5%. The NLC powder was gathered in a collector, sealed in an aluminum polyethylene (PE) liner bag, and stored in a desiccator at room temperature for further studies.

### 3.5. NLCs Characterization Techniques

#### 3.5.1. Hydrodynamic Diameter and Zeta Potential of NLCs

The hydrodynamic diameter, PDI, and zeta potential of both loaded NLCs and redispersed dried NLCs were evaluated using dynamic light scattering (DLS) analysis with a Zetasizer ZS (Malvern Panalytical, Malvern, UK). The loaded NLCs were dispersed in DI water and analyzed immediately after preparation, whereas the spray-dried NLCs were redispersed in DI water prior to testing. Each sample was examined in triplicate, and the average values for diameter, PDI, and zeta potential, along with their standard deviations, were calculated.

#### 3.5.2. Morphology of Spray Dried NLCs

The morphological characteristics of the spray-dried NLCs were examined using a scanning electron microscope (SEM). The samples were mounted on stubs and coated with a thin layer of gold to enhance visualization. This analysis was performed with a Philips XL 30 ESEM instrument (FEI Company, Hillsboro, OR, USA) at magnifications of 10,000× and 15,000×.

#### 3.5.3. Particle Size and Distribution of Spray Dried NLCs

The particle size and distribution of the spray-dried NLCs were measured in triplicate using a Mastersizer 3000 Ultra (Malvern Panalytical, Malvern, UK) with an Aero S dry powder dispersion unit. An appropriate amount of powder was placed into the hopper with a 1.5 mm gap. The obscuration ranged from 0.1% to 10%, with a feed rate of 50% and air pressure set at 2.5 barg. The data were expressed as d (4,3), representing the volume-weighted mean diameter. The particle size distribution, analyzed by the Mastersizer Xplorer software (version 5.20.2410.072), was reported in terms of span.

### 3.6. In Vitro Release

The curcumin release study was modified from [66]. Dialysis bags with a molecular weight cutoff of 12,000 Da (Merck, Rahway, NJ, USA) were utilized to evaluate curcumin release from NLC formulations. The sacks were pre-soaked in distilled water overnight prior to application. Each dialysis sack was filled with NLCs and simulated gastric medium (SGM). The SGM consisted of 0.32% pepsin, 2 g NaCl, and 7 mL of 37% HCl, with the pH adjusted to 2.0 using 1.0 M HCl. The sacks were securely tied to prevent leakage and subsequently placed in a beaker containing methanol for incubation at 37 °C with stirring at 50 rpm using a magnetic bar. At predetermined intervals (0, 2, 4, 6, 8, and 10 h), 1 mL of methanol was withdrawn and replaced with an equal volume. The contents of the dialysis sacks were then centrifuged at 10,000 rpm at 4 °C for 30 min. The precipitate was transferred to another dialysis sack containing simulated intestinal fluid, which included 0.4 mg/mL of lipase, 0.7 mg/mL of bile extract solution, 0.5 mg/mL of pancreatin, and 1 mL of 750 mM CaCl_2_ solution, with the pH adjusted to 7 using 0.1 M NaOH. Similar to the gastric phase, the sacks were placed in a beaker containing methanol and incubated at 37 °C while stirring. At the same predetermined intervals, 1 mL of methanol was withdrawn and replaced. The released curcuminoids in methanol were quantified using HPLC. Briefly, the standard curcumin solutions were prepared in the concentration range of 20–100 µg/mL. The standard curve and HPLC chromatogram of curcumin are shown in Figures S3 and S4. Regarding the HPLC procedure, Agilent HPLC and a ZORBAX Eclipse Plus C18 analytical column (4.6 mm × 250 mm; 5 µm particle size, Agilent, Cheshire, UK) were operated at 45 °C. Acetonitrile served as solvent A, while formic acid (1% *v*/*v*) in DI water served as solvent B for the gradient elution as follows: 0–12 min, 10% A; 12–22, 60% A; 22–30 min, 10% A with a constant flow rate of 1.0 mL/min. The filtered sample (10 μL) was injected into the column and detected for the presence of curcumin at a UV wavelength of 425 nm. DDSolver^®^ software [67], a menu-driven add-in program for Microsoft Excel written in Visual Basic for Applications was used to evaluate the kinetic model fitting of the release profiles. The coefficients of determination (R^2^) and the AIC were used to assess model fitting.

### 3.7. Stability Property Determination [68]

Turmeric extract, chili extract, a mixture of turmeric, coffee, and chili extracts (mixed extract), and mixed extracts in NLCs formulations were evaluated for stability properties under stress conditions compared with mixed standards of curcumin, chlorogenic, and capsaicin (mixed standard). The stock solution of all test compounds was prepared in methanol. For the acid, base, and oxidation studies, the stock solution was transferred to the volumetric flask and treated with acid (1 N HCl), base (1 N NaOH), or peroxide (30% H_2_O_2_) solution before being adjusted to the volume, protected from light and heating at 60 °C for 2 h. For the thermal stability study, the stock solution was transferred to the volumetric flask, adjusted the volume with methanol, protected from light, and then heated at 60 °C for 2 h. During the photostability study, the stock solution was transferred to the volumetric flask, the volume was adjusted with methanol, and the solution was exposed to white fluorescent light for 6 h. The test compounds subjected to the stress test were prepared at a final concentration of 60 µg/mL equivalent to curcumin and capsaicin. After complete treatment, all test solutions were filtrated through a syringe filter before analysis by HPLC. The ANOVA test was performed by GraphPad Prism version 025, (GraphPad Software Inc., San Diego, CA, USA).

## 4. Conclusions

The combination of turmeric, coffee, and chili extracts underwent a thorough study to determine the most effective antioxidant ratio and concentration, as obtained from a DoE approach, which was successfully validated at concentration ratio of 1400:6000:8000 µg/mL. The study revealed that the mixed extract exhibited higher antioxidant activity compared with each single extract. Subsequently, the mixed extract was evaluated for its cytotoxic and anti-inflammatory activities through the nitric oxide assay and inflammatory-related gene expressions. The use of the mixed extract effectively reduced the secretion of pro-inflammatory cytokines at a lower concentration than turmeric extract alone, enhancing its safety profile while maintaining equivalent efficacy. The mixed extract, at a concentration of 14.16 mg/dose, was used to formulate NLCs and successfully prepared with a nanosized hydrodynamic diameter (258.0 ± 10.2 nm). It also demonstrated an ability to redisperse after the spray-drying process. The stability of the formulation under various stress conditions was evaluated using curcumin and capsaicin as marker compounds. Capsaicin was proven to be more stable than curcumin under all stressed conditions in the NLCs formulation. Interestingly, curcumin in the NLC formulation enhanced stability under basic conditions. The main curcumin release mechanism from NLCs was a combination of Fickian diffusion and Case-II transport. Based on these achievements, the mixed extract showed potential for use as a stable and sustained-release nutrition supplement for combating inflammation-related syndromes. There are some considerations to note regarding this study. First, the sources of the extracts should be validated to ensure consistency in the content of key active compounds, including curcumin, capsaicin, chlorogenic acid, and caffeine, before their use in formulation preparation. Additionally, the ratio of the mixed extracts was slightly adjusted to address practical challenges encountered during the formulation process. These considerations will be addressed in future studies to further optimize the preparation and standardization of the formulation. Our next phase will involve an in vivo study of this combination and explore other techniques, such as solid dispersion, to enhance the release properties and stability of this mixed extract.

## Figures and Tables

**Table 1:** Full factorial design runs of turmeric extract, chili extract, and coffee extract concentrations generated from the Design Expert software with percentage inhibition from DPPH-RSA assay and lipid peroxidation inhibition assay.

Run	Concentration (µg/mL)			DPPH-RSA Assay	Lipid Peroxidation Inhibition Assay
	Turmeric Extract	Coffee Extract	Chili Extract	% Inhibition	% Inhibition
1	1400	6000	8000	93.29 ± 0.51	46.69 ± 2.31
2	770	33,000	4400	93.15 ± 1.46	39.55 ± 3.56
3	140	6000	8000	84.18 ± 0.54	41.88 ± 5.40
4	770	33,000	8000	89.85 ± 1.20	50.86 ± 1.96
5	1400	60,000	8000	93.94 ± 5.48	55.85 ± 3.45
6	770	33,000	800	90.27 ± 1.22	45.45 ± 4.96
7	140	33,000	4400	88.90 ± 0.83	44.78 ± 3.90
8	1400	6000	800	92.03 ± 0.39	44.65 ± 5.12
9	770	60,000	4400	91.09 ± 0.68	49.27 ± 1.53
10	770	33,000	4400	90.13 ± 2.21	37.99 ± 4.72
11	770	33,000	4400	97.34 ± 0.65	36.94 ± 0.47
12	140	60,000	8000	89.63 ± 1.56	41.43 ± 5.47
13	1400	33,000	4400	91.76 ± 1.02	44.78 ± 3.32
14	1400	60,000	800	95.87 ± 1.67	34.85 ± 3.37
15	770	6000	4400	90.51 ± 0.75	48.52 ± 3.32
16	140	60,000	800	89.16 ± 0.22	19.56 ± 1.24
17	140	6000	800	63.90 ± 0.79	6.07 ± 3.14
Control (Trolox) *				94.59 ± 0.05	71.39 ± 0.24

* Trolox concentrations used in DPPH-RSA and lipid peroxidation inhibition assays were 25 and 1000 µg/mL, respectively.

**Table 2:** Comparison between experimental values and prediction values of percentage inhibition of turmeric extract, coffee extract, and chili extract at concentrations of 1400:6000:8000 µg/mL.

Antioxidant Methods (Unit)	Experimental Value	Predicted Value	Residual of Response
DPPH-RSA assay (% inhibition)	92.13 ± 1.47	93.78	1.65
Lipid peroxidation inhibition assay (% inhibition)	46.08 ± 3.47	55.60	9.52

**Table 3:** IC_50_ values of coffee extract, turmeric extract, chili extract, and mixed extract from MTT assay of RAW264.7 cells.

Sample	IC_50_ (µg/mL)
Coffee extract	858.99 ± 4.95
Turmeric extract	34.99 ± 2.18
Chili extract	211.93 ± 2.69
Mixed extract	122.53 ± 8.94

**Table 4:** Amounts of turmeric extract, coffee extract and chili extract and their active ingredient contents per day.

	Amount of Extract (mg)	Active Ingredient Content per Day
Turmeric extract	1.77	Curcumin 1.16 mg
Coffee extract	5.31	Chlorogenic acid 27 µg Caffeine 2.7 µg
Chili extract	7.08	Capsaicin 0.9 mg
Total	14.16	

## Data Availability

The original contributions presented in this study are included in the article/Supplementary Material. Further inquiries can be directed to the corresponding author.

## Supplementary Materials

The following supporting information can be downloaded at: https://www.mdpi.com/article/10.3390/plants14020236/s1.
