# Supplementary material for: Boosting Therapeutic Effect of Turmeric, Coffee, and Chili Extracts Through Experimental Design and Encapsulation as Nanostructured Lipid Carriers for Novel Heath Supplements
Source: Plants (Basel). 2025 Jan 16;14(2):236. doi: 10.3390/plants14020236 (PMC11768308; doi:10.3390/plants14020236)
Supplement: Supplementary file 1 [file plants-14-00236-s001.zip › plants-3418444-supplementary.pdf]

Supplementary Data

Boosting Therapeutic Effect of Turmeric, Coffee, and Chili Extracts Through Experimental Design and Encapsulation as Nanostructured Lipid Carriers for Novel Heath Supplements

Pratchaya Tipduangta [1], Phennapha Saokham [1], Jutamas Jiaranaikulwanitch [1], Siriporn Okonogi [1,2], Chadarat Ampasavate [1,2] and Kanokwan Kiattisin [1,*]

[1] Department of Pharmaceutical Sciences, Faculty of Pharmacy, Chiang Mai University, Chiang Mai 50200, Thailand; pratchaya.t@cmu.ac.th (P.T.); phennapha.s@cmu.ac.th (P.S.); jutamas.jia@cmu.ac.th (J.J.); siriporn.o@cmu.ac.th (S.O.); chadarat.a@cmu.ac.th (C.A.)

[2] Center of Excellence in Pharmaceutical Nanotechnology, Faculty of Pharmacy, Chiang Mai University, Chiang Mai 50200, Thailand

* Correspondence: kanokwan.k@cmu.ac.th

Supplementary Figures

1. **Supplementary Figure S1** Relationship of extracts concentration [horizontal axis] and antioxidation response [vertical axis] as % inhibition analyzed from the design expert software and DPPH and lipid peroxidation assay of individual and mix extract (A) relationship between turmeric extract concentration and % DPPH inhibition (B) relationship between turmeric extract concentration and % lipid peroxidation inhibition (C) relationship between chili extract concentration and % lipid peroxidation inhibition

2. **Supplementary Figure S2** (A) Hydrodynamic diameter of NLCs, (B) PDI of NLCs. and (C) Zeta potential of NLCs.

3. **Supplementary Figure S3** Standard curve of curcumin using HPLC

4. **Supplementary Figure S4** HPLC chromatogram of curcumin (60 µg/mL) at 425 nm

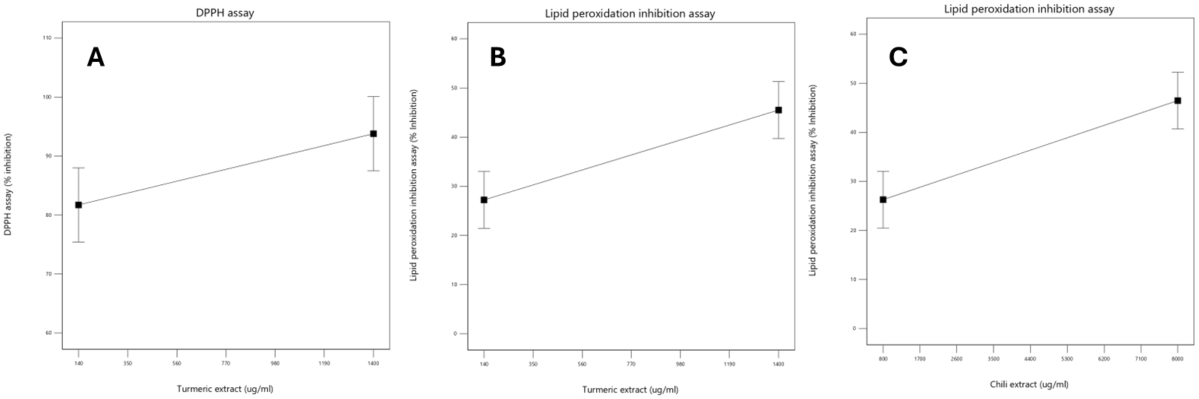

Supplementary Figure S1 Relationship of extracts concentration [horizontal axis] and antioxidation response [vertical axis] as % inhibition analyzed from the design expert software and DPPH and lipid peroxidation assay of individual and mix extract (A) relationship between turmeric extract concentration and % DPPH inhibition (B) relationship between turmeric extract concentration and % lipid peroxidation inhibition (C) relationship between chili extract concentration and % lipid peroxidation inhibition

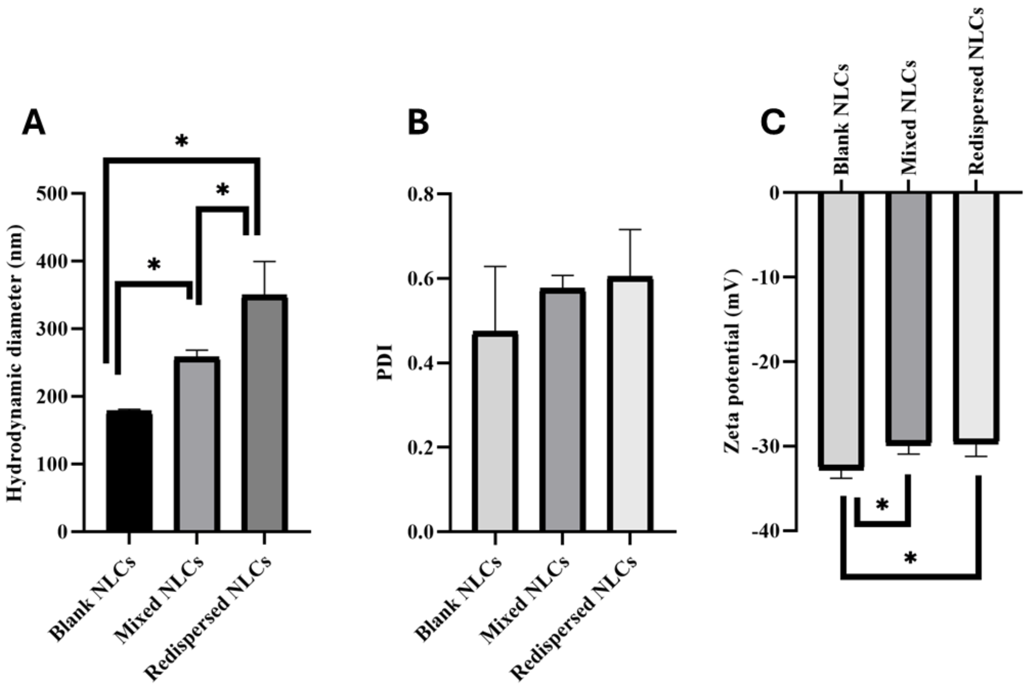

Supplementary Figure S2 (A) Hydrodynamic diameter of NLCs, (B) PDI of NLCs. and (C) Zeta potential of NLCs.

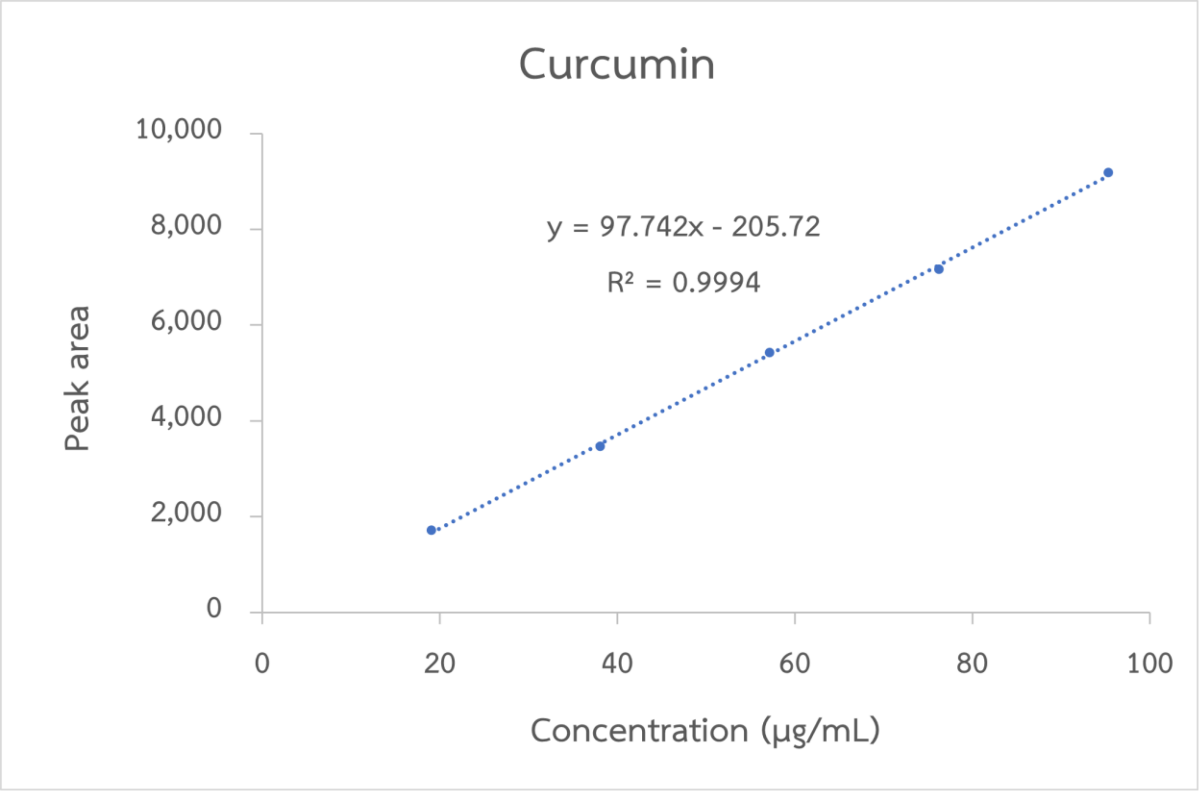

Supplementary Figure S3 Standard curve of curcumin using HPLC

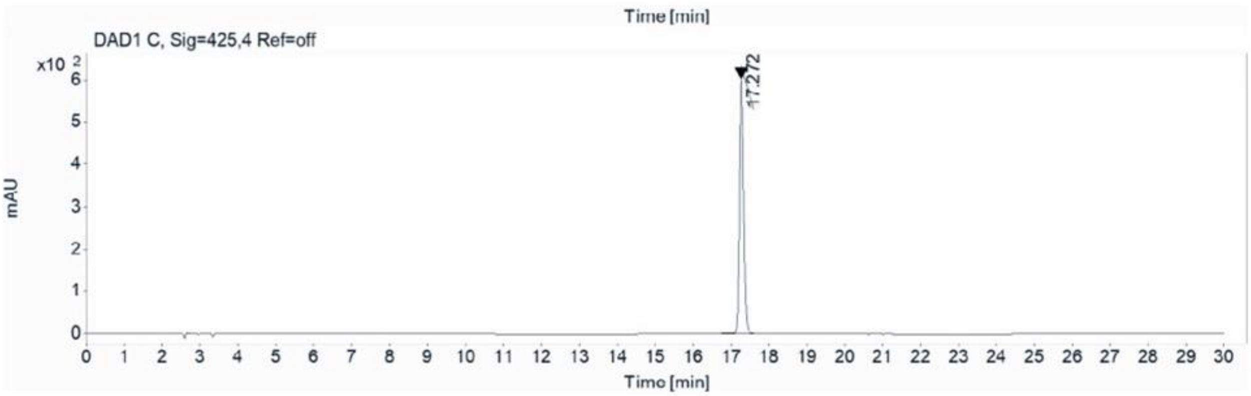

Supplementary Figure S4 HPLC chromatogram of curcumin at 425 nm

Supplementary Table

1. **Supplementary Table S1** IC_{50} value of each extract by DPPH and lipid peroxidation inhibition assays
2. **Supplementary Table S2** Lower limit and upper limit of extract concentration used in the DoE experiments
3. **Supplementary Table S3** ANOVA analysis showing relationship between Turmeric extract concentration to %inhibition on the DPPH assay

Supplementary Table S1 IC_{50} value of each extract by DPPH and lipid peroxidation inhibition assays

Extract	DPPH assay (IC_{50} µg/ml)	Lipid peroxidation inhibition assay (IC_{50} mg/ml)
**Turmeric extract**	13.66 ± 0.67	0.79 ± 0.01
**Coffee extract**	576.65 ± 10.88	ND
**Chili extract**	334.13 ± 18.04	3.22 ± 0.49

ND mean not detectable.

Supplementary Table S2 Lower limit and upper limit of extract concentration used in the DoE experiments

Extract	Lower limit µg/ml	Upper limit µg/ml
**Turmeric extract**	140	1400
**Coffee extract**	6000	60000
**Chili extract**	800	8000

Supplementary Table S3 ANOVA analysis showing relationship between Turmeric extract concentration to %inhibition on the DPPH assay

Source	Sum of Squares	df	Mean Square	F-value	p-value
A-Turmeric extract	291.13	1	291.13	4.9	0.0578*
Curvature	73.15	1	73.15	1.23	0.2994
Lack of Fit	449.13	6	74.86	5.71	0.1566

* p-value < 0.10
